# Rationally Engineered D-Amino Acid Peptide DT7-3 Combats Multidrug-Resistant *Helicobacter pylori* via a Novel “Triple-Hit” Mechanism

**DOI:** 10.3390/microorganisms14040744

**Published:** 2026-03-26

**Authors:** Shiying Yan, Xin Yan, Jiarui Zhao, Yue Zhou, Changyi Huang, Yiping Chen, Jia Wang, Jian Zhang, Chaoyi Han, Yu Gao, Tianlan Jiang, Hansheng Zhu, Hao Shi, Fosheng Li, Jian Zhao, Mei Cao

**Affiliations:** 1 Key Laboratory of Biological Resource and Ecological Environment of Chinese Education Ministry, College of Life Sciences, Sichuan University, Chengdu 610064, China; 13107178792@163.com (S.Y.);; 2 Animal Disease Prevention and Green Development Key Laboratory of Sichuan Province, College of Life Sciences, Sichuan University, Chengdu 610064, China; 3 Core Laboratory, Sichuan Provincial People’s Hospital, School of Medicine, University of Electronic Science and Technology of China, Chengdu 610072, China

**Keywords:** *Helicobacter pylori*, multidrug resistance, antimicrobial peptide, mechanism of action, D-peptide

## Abstract

*Helicobacter pylori* (*H. pylori*) is the primary etiological agent for chronic gastritis, peptic ulcers, and gastric adenocarcinoma. The alarming rise in multidrug-resistant (MDR) strains, particularly against clarithromycin (CLR), metronidazole (MNZ), and levofloxacin (LVX), has severely compromised standard therapies. Thus, there is an urgent clinical need for novel antimicrobial agents that operate through distinct mechanisms to bypass resistance pathways and mitigate gastric cancer risk. We designed and synthesized a series of antimicrobial peptides, focusing on the proteolytically stable all-D-amino acid enantiomer, DT7-3, derived from a probiotic-sourced template. Minimum inhibitory concentrations (MICs) were determined against standard strains and 11 clinical MDR isolates via the broth microdilution method. Antimicrobial mechanisms were elucidated using scanning electron microscopy (SEM) for morphology, fluorescence-based assays for anti-adhesion activity, and real-time qPCR to quantify virulence gene expression (*babA*, *ureA*, and *vacA*). Biocompatibility was assessed using defibrinated sheep erythrocytes, gastric epithelial cells (GES-1), and representative beneficial gut microbiota. Analysis of the clinical isolates revealed resistance rates of 63.6% for CLR/LVX and 81.8% for MNZ, with 54.5% identified as MDR. DT7-3 exhibited superior potency (MIC 1–32 µg/mL) against all strains, significantly outperforming its L-enantiomer counterparts. Mechanistic studies unveiled a “triple-hit” mechanism: (1) rapid membrane disruption; (2) potent inhibition of bacterial adhesion to host cells (~60% reduction at 0.5 × MIC); (3) significant downregulation of critical virulence factors (*babA*, *ureA*, and *vacA*). Furthermore, DT7-3 showed an excellent safety profile, with negligible hemolysis (<5% at 32 µg/mL) and minimal cytotoxicity toward GES-1 cells, yielding a high selectivity index (SI, MHC/MIC) > 32 relative to mammalian cells. Crucially, DT7-3 showed high selectivity for the pathogen over beneficial gut microbiota (MIC > 128 µg/mL, SI > 16). Crucially, DT7-3 maintained potent bactericidal activity (MIC ≤ 16 µg/mL) even under cholesterol-enriched conditions. The engineered D-peptide DT7-3 is a potent candidate for combating MDR *H. pylori*. Its multifaceted mechanism, targeting bacterial viability while suppressing core virulence factors, positions it as a robust lead compound for next-generation eradication therapies aimed at reducing the burden of *H. pylori*-associated diseases.

## 1. Introduction

*Helicobacter pylori* (*H. pylori*) is a Gram-negative, microaerophilic bacterium that colonizes the gastric epithelium of approximately half the global population [1,2]. Persistent infection is the primary etiological factor for a spectrum of gastric pathologies, ranging from chronic gastritis and peptic ulcers to gastric adenocarcinoma and mucosa-associated lymphoid tissue (MALT) lymphoma [2,3]. As a Class I carcinogen designated by the World Health Organization (WHO), *H. pylori* represent a significant public health burden [2]. The pathogen’s ability to establish chronic infection relies on a complex interplay of virulence factors: urease neutralizes gastric acidity to facilitate colonization; flagella drive motility through the mucus layer; and adhesins (e.g., BabA, SabA) mediate tight attachment to the gastric epithelium, preventing clearance and delivering toxins such as CagA and VacA directly to host cells [3,4].

Currently, the cornerstone of *H. pylori* management relies on combination therapies involving a proton pump inhibitor (PPI) and two or more antibiotics, typically clarithromycin (CLR), amoxicillin (AMX), or metronidazole (MNZ) [1,5]. However, the clinical landscape has shifted dramatically over the past decade. The widespread and often indiscriminate use of antibiotics has fueled a global surge in resistance, particularly to CLR and MNZ, compromising the efficacy of standard regimens [3,5]. In many regions, eradication rates have plummeted below 80%, leading to recurrent treatment failures and increased healthcare costs. Recognizing this crisis, the WHO has categorized CLR-resistant *H. pylori* as a high-priority pathogen requiring urgent research and development of new therapeutic strategies [1]. Therefore, identifying novel agents that can circumvent traditional resistance mechanisms is not merely advantageous but essential.

In this context, antimicrobial peptides (AMPs) have garnered significant attention as a potential alternative to conventional antibiotics. As ancient components of the innate immune system, AMPs offer a distinct set of advantages: they typically exhibit broad-spectrum activity, rapid bactericidal kinetics, and, most importantly, a low propensity for inducing resistance [6,7]. Unlike traditional antibiotics that primarily work by inhibiting essential bacterial processes, such as cell wall synthesis, protein synthesis, nucleic acid synthesis, or specific metabolic pathways—most cationic AMPs act by electrostatically targeting the negatively charged bacterial membrane and physically disrupting its integrity [7]. This “physical” mode of action makes it evolutionarily difficult for bacteria to develop resistance via single-point mutations.

The rational design of AMPs hinges on understanding structure–activity relationships (SARs), a fundamental principle guiding peptide engineering [8]. Among the critical physicochemical parameters, net positive charge is paramount; a sufficient positive charge (typically +2 to +9) facilitates the initial electrostatic attraction to the anionic bacterial surface, particularly the lipopolysaccharide (LPS)-rich outer membrane of *H. pylori* [9]. Furthermore, hydrophobicity and amphipathicity govern the peptide’s ability to insert into and disrupt the lipid bilayer core. Upon membrane binding, most lytic AMPs fold into an amphipathic α-helical structure, partitioning their hydrophobic face into the membrane while exposing the hydrophilic face [8,9]. Building on these principles, structural modification, such as enhancing cationic charge or introducing bulky hydrophobic residues like tryptophan, has proven effective in boosting antimicrobial potency.

However, a major bottleneck restricting the clinical translation of natural AMPs is their inherent instability. Composed of L-amino acids, natural peptides are susceptible to rapid degradation by host proteases, particularly pepsin in the gastric environment [10]. To overcome this hurdle, the incorporation of D-amino acids (enantiomers) has emerged as a robust strategy. D-peptides retain the physicochemical properties and membrane-targeting ability of their L-counterparts but are resistant to proteolytic cleavage, thereby significantly extending their half-life and therapeutic potential in vivo [11,12]. In summary, combining rational sequence optimization with chiral modification represents a promising avenue to develop potent and stable peptide therapeutics [9,10].

In this study, we sought to address the dual challenges of drug resistance and peptide stability. We started with a parental peptide, T7, identified from the fermentation supernatant of *Lacticaseibacillus casei* T1. Our previous research has established that *Lacticaseibacillus casei* T1 can effectively colonize the gastric mucosa and attenuate *H. pylori*-induced inflammation and gut microbiota disorders in mice [13]. Through rational design, we engineered two derivatives: T7-3, optimized for enhanced membrane interaction via charge and hydrophobicity modifications, and DT7-3, an all-D-amino acid enantiomer designed for stability. We systematically evaluated their efficacy against a panel of clinical MDR-*H. pylori* isolates and elucidated their mechanisms of action, focusing on membrane disruption, anti-adhesion effects, and virulence gene suppression, while quantitatively assessing their selectivity relative to host cells and representative beneficial gut microbiota.

## 2. Materials and Methods

### 2.1. Peptide and Synthesis

Peptides were synthesized via standard Fmoc solid-phase peptide synthesis (SPPS) methods using 2-chlorotrityl chloride resin (0.15 mmol scale, GL Biochem, Shanghai, China). Coupling reactions were performed using Fmoc-D-amino acids (3 equiv), DIC (3 equiv), and HOBt (3 equiv) in DMF. All synthesis reagents and solvents were purchased from Sigma-Aldrich, St. Louis, MO, USA. Fmoc deprotection was carried out using 20% piperidine in DMF (*v*/*v*). Upon completion of the synthesis, peptides were cleaved from the resin using a cocktail of TFA/H_2_O/EDT/TIS (95:1:2:2, *v*/*v*/*v*/*v*) for 2 h. The crude peptides were precipitated in cold diethyl ether, centrifuged, and lyophilized.

Purification was performed using a Shimadzu HPLC-20AD system (Shimadzu, Kyoto, Japan) equipped with a Daisogel C18 column (20 mm × 250 mm, 8 µm, DAISOGEL, Osaka, Japan). Elution was achieved with a linear gradient of water/acetonitrile containing 0.1% TFA (10%~55% acetonitrile over 40 min) at a flow rate of 10 mL/min. The molecular weight and purity (>95%) of the synthesized peptides were confirmed using a Shimadzu LCMS-2020 system (Shimadzu, Kyoto, Japan). For experimental use, the lyophilized peptide powders were dissolved in dimethyl sulfoxide (DMSO, Sigma-Aldrich, St. Louis, MO, USA) to prepare a 1000 µg/mL stock solution and stored at −80 °C until use.

### 2.2. Bacterial Strains and Cell Lines

Between September 2020 and January 2021, 11 *H. pylori* strains were isolated from patients diagnosed with various gastric diseases at the Department of Gastroenterology, Sichuan Provincial People’s Hospital. The strains were cultured on Columbia agar plates supplemented with 5% defibrinated sheep blood (Solarbio, Beijing, China) under microaerophilic conditions (5% O_2_, 10% CO_2_, and 85% N_2_) for 3–4 days. The identification of *H. pylori* was confirmed by assessing colony morphology, Gram staining, and positive biochemical reactions (oxidase, catalase, and urease), followed by 16S rRNA gene sequencing to confirm their identity as *H. pylori* (ethical review approved by the Ethics Committee) [14]. The standard strains *H. pylori* SS1 (ATCC 43501), *H. pylori* 26695 (ATCC 700392) and the multidrug-resistant *Staphylococcus aureus* (MRSA) strain SCQL1 were conserved by the Key Laboratory of Resources Microbiology and Biotechnology of Sichuan Province, Sichuan University. All isolates were subsequently preserved at −80 °C in sterile brain heart infusion (BHI) broth (Oxoid, Basingstoke, UK) containing 20% glycerol for further analysis. Additionally, the human gastric mucosal epithelial cell line GES-1 (conserved by the Key Laboratory, Sichuan University) was used for selectivity assays. 

### 2.3. Characterization of Peptides

Key physicochemical properties were calculated using the HeliQuest analysis tool (https://heliquest.ipmc.cnrs.fr/cgi-bin/ComputParams.py, accessed on 15 February 2025). Helical wheel projections were generated to visualize amphipathicity using the NetWheels web application (http://www.lbqp.unb.br/NetWheels/, accessed on 23 March 2026). Tertiary (3D) structural models were predicted using the I-TASSER server (https://zhanggroup.org/, accessed on 14 August 2025), andmolecular structural formulas were generated via NovoPro PepSMI (https://www.novoprolabs.com/tools/convert-peptide-to-smiles-string, accessed on 7 October 2025) [15]. CL plasma penetration, LogP and LogD7.4 were calculated using ADMETlab 3.0 (https://admetlab3.scbdd.com/server/screening, accessed on 18 February 2026) [16].

### 2.4. Antibiotic Susceptibility Testing (MICs)

To assess the baseline resistance of our clinical cohort, the Minimum Inhibitory Concentrations (MICs) of five standard antibiotics, amoxicillin (AMX), clarithromycin (CLR), levofloxacin (LVX), tetracycline (TET), and metronidazole (MNZ) (all antibiotics purchased from Sigma-Aldrich, St. Louis, MO, USA) were determined using the agar dilution method, strictly following Clinical and Laboratory Standards Institute (CLSI) guidelines [17,18,19]. The resistance breakpoints were defined as: CLR ≥ 1 µg/mL, LVX ≥ 2 µg/mL, and MNZ ≥ 8 µg/mL. The antibacterial activity of peptides (T7, T7-3, DT7-3) was evaluated using the broth microdilution method. Peptides were prepared using a two-fold serial dilution method in sterile BHI broth, with final concentrations ranging from 128–0.125 µg/mL. Briefly, bacterial suspensions were adjusted to 1 × 10^6^ CFU/mL in BHI broth supplemented with 10% fetal bovine serum (FBS, Gibco, Grand Island, NY, USA). Serial dilutions of peptides were added to 96-well plates. After 72 h of microaerophilic incubation at 37 °C, the MIC was defined as the lowest concentration preventing visible growth.

To evaluate the impact of host-derived membrane cholesterol on antimicrobial efficacy, MIC assays were performed using media supplemented with water-soluble cholesterol (Sigma-Aldrich, St. Louis, MO, USA) at concentrations of 50 and 100 µg/mL. *H. pylori* strains (SS1, 26695, and SCU-HP-210106A) were pre-incubated in cholesterol-enriched Brucella broth (Oxoid, Basingstoke, UK) for 12 h under microaerophilic conditions. This pre-incubation allowed the bacteria to actively sequester and glucosylate cholesterol into their lipid bilayers prior to peptide treatment, following established protocols [20]. Subsequent MIC determinations were conducted using the standard broth microdilution method in the corresponding cholesterol-supplemented media.

### 2.5. Mechanism of Action Studies

#### 2.5.1. Scanning Electron Microscopy (SEM)

To visualize peptide-induced morphological changes, *H. pylori* strain SCU-HP-210106A (1 × 10^7^ CFU/mL) was treated with peptides at 1× MIC for 1 h at 37 °C [21]. Cells were harvested, washed with PBS, and fixed with 2.5% glutaraldehyde (Sigma-Aldrich, St. Louis, MO, USA). Samples were then dehydrated through a graded ethanol series, critical-point dried, sputter-coated with gold, and examined using a scanning electron microscope (Helios G4 UC, Thermo Fisher Scientific, Waltham, MA, USA).

#### 2.5.2. Anti-Adhesion Assay

Adhesion is a prerequisite for *H. pylori* colonization. We evaluated the peptides’ ability to inhibit this step using a fluorescence-based assay [22]. GES-1 cells were seeded in 96-well plates and grown to confluence. *H. pylori* (SCU-HP-210106A) were labeled with fluorescein isothiocyanate (FITC, Sigma-Aldrich, St. Louis, MO, US). The labeled bacteria were pre-incubated with sub-inhibitory concentrations of peptides (0.125–4 µg/mL) for 30 min before being added to the GES-1 monolayers. After co-incubation, non-adherent bacteria were washed away, and fluorescence intensity was measured.

#### 2.5.3. Real-Time Quantitative PCR (qPCR)

To investigate the molecular basis of virulence suppression, *H. pylori* (SCU-HP-210106A) were treated with sub-MICs of T7-3 or DT7-3 for 6 h [23,24]. Total RNA was extracted using TRIzol reagent (Invitrogen, Carlsbad, CA, USA). cDNA was synthesized using a Reverse Transcription Kit (Takara Bio, Kusatsu, Japan). Quantitative PCR was performed using SYBR Green Master Mix (Takara Bio, Kusatsu, Japan). The relative expression levels of virulence genes (*babA*, *katA*, *ureA*, *vacA*) were calculated using the 2^−ΔΔCt^ method, with 16S rRNA serving as the internal control. Primers sequences were shown in Table S1 of Supplementary Materials.

### 2.6. Biocompatibility and Selectivity

#### 2.6.1. Hemolysis Assay

Hemolytic activity was assessed using fresh defibrinated sheep erythrocytes (4%, *v*/*v*) in PBS [25,26]. These erythrocytes (RBCs) were incubated with peptides (1–32 µg/mL) for 1 h at 37 °C. Triton X-100 (1%) and PBS were used as positive and negative controls, respectively. After incubation and centrifugation, the absorbance of the supernatant (A) was measured at 540 nm. The percentage of hemolysis was calculated by normalizing the absorbance of the peptide-treated sample (*A_sample_*) against the positive (*A_positive_*) and negative (*A_negative_*) controls using the following equation: (*A_sample_* − *A_negative_*)/(*A_positive_* − *A_negative_*)) × 100%. This ensures that the data accurately reflect the relative lytic potency of each peptide.

#### 2.6.2. Cytotoxicity Assay

The cytotoxicity of DT7-3 and its derivatives toward human gastric mucosal epithelial cells (GES-1) was evaluated using the CCK-8 assay (Biosharp, Hefei, China) [27,28]. Briefly, cells (5 × 10^3^ cells/well) were seeded in 96-well plates and incubated for 24 h, followed by treatment with peptides (1–128 ug/mL) for an additional 1–48 h. Cell viability was quantified by measuring the absorbance at 450 nm using a microplate reader (Synergy H1, BioTek Instruments, Winooski, VT, USA) after a 2 h incubation with CCK-8 reagent and expressed as a percentage relative to the untreated control group.

#### 2.6.3. Gut Microbiota Susceptibility Assay

The susceptibility of representative beneficial gut microbiota strains (*Lacticaseibacillus casei* T1, *Bifidobacterium longum* J5, and *Lactiplantibacillus plantarum* N1) to the peptides was determined using the broth microdilution method in MRS broth (Oxoid, Basingstoke, UK), following CLSI guidelines [29,30]. Briefly, overnight cultures were diluted to a final inoculum of approximately 5 × 10^5^ CFU/mL and incubated with serial twofold dilutions of the peptides (1 to 128 µg/mL) in 96-well plates. Peptide-free wells served as the growth control. Plates were incubated at 37 °C for 24 h; *Bifidobacterium* strains were incubated under anaerobic conditions, while *Lacticaseibacillus* strains were incubated microaerophilically (5% CO_2_). The MIC was defined as the lowest peptide concentration that completely inhibited visible turbidity.

#### 2.6.4. Evaluation of Selectivity Index (SI)

To quantitatively evaluate the cell-type specificity and therapeutic window of the peptides, the Selectivity Index (SI) was calculated using the following formula: SI = MHC/MIC [31]. The Minimum Hemolytic Concentration (MHC) was defined as the lowest peptide concentration that caused 10% hemolysis (*HC*_10_) [32]. For cases where no significant hemolysis or gut microbiota inhibition was observed at the highest tested concentrations, the MHC or *MIC_gut microbiota_* was conservatively estimated as the maximum concentration used in the assay for SI calculation purposes.

### 2.7. Statistical Analysis

All experiments were performed in triplicate. Data are expressed as mean ± standard deviation (SD). Statistical differences were analyzed using Student’s *t*-test or one-way ANOVA followed by Dunnett’s test (GraphPad Prism 8.0, San Diego, CA, USA). *p* < 0.05 was considered statistically significant.

## 3. Results

### 3.1. Design, Synthesis and Characterization of Peptides

**Figure 1:**
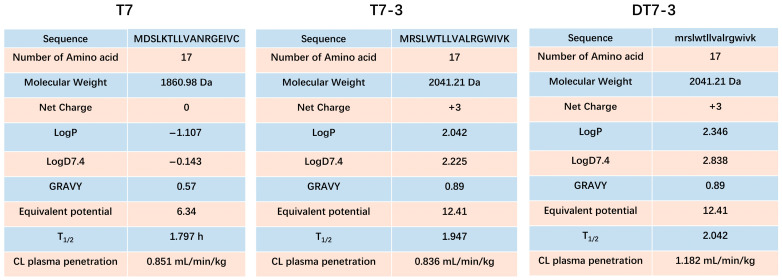
Sequences and key physicochemical parameters of T7 derived peptides.

The parental peptide T7 (Figure 1), derived from *Lacticaseibacillus casei* T1 metabolite, served as the scaffold for this study [13,33]. Our design strategy focused on overcoming the limitations of natural peptides. For T7-3, we introduced specific mutations: acidic (D) and neutral (C) residues were replaced with basic residues (R, K) to increase net positive charge (+1 to +3), enhancing electrostatic attraction to bacterial membranes. Furthermore, Tryptophan (W) residues were introduced to act as membrane anchors, and hydrophobicity was optimized to promote amphipathic helix formation. To address proteolytic instability, the DT7-3 variant was synthesized as a D-enantiomer of T7-3.

The candidate peptides (T7, T7-3, and DT7-3) were successfully synthesized using the standard Fmoc solid-phase peptide synthesis (SPPS) strategy. Following purification by RP-HPLC, analytical chromatograms demonstrated that all synthesized peptides achieved a high purity of greater than 95%. Furthermore, the molecular identities were verified by LC-MS analysis, where the observed molecular weights were in excellent agreement with the theoretical values (Figure S1), confirming the successful preparation of the target peptides for subsequent biological evaluation.

Antimicrobial peptides (AMPs) typically compromise bacterial membrane integrity by leveraging electrostatic attraction to anionic surfaces followed by hydrophobic insertion. Predictive modeling using ADMET 3.0 revealed that while T7-3 and DT7-3 possess cationic properties (+3 net charge at pH 7.4), the engineered D-enantiomer DT7-3 exhibits optimized lipophilicity (LogP = 2.346; GRAVY = 0.89) compared to T7 (LogP = −1.107) and T7-3 (LogP = 2.042), traits conducive to enhanced membrane interaction. Furthermore, the favorable pharmacokinetic profiles obtained via chiral-sensitive modeling demonstrated that DT7-3 possesses a superior predicted half-life (T1/2 = 2.042 h) and a plasma clearance rate of 1.182 mL/min/kg, suggesting promising potential for in vivo delivery. In contrast, the L-enantiomer counterparts T7 and T7-3 exhibited lower predicted half-lives of 1.797 h and 1.947 h, respectively (Figure 1).

**Figure 2:**
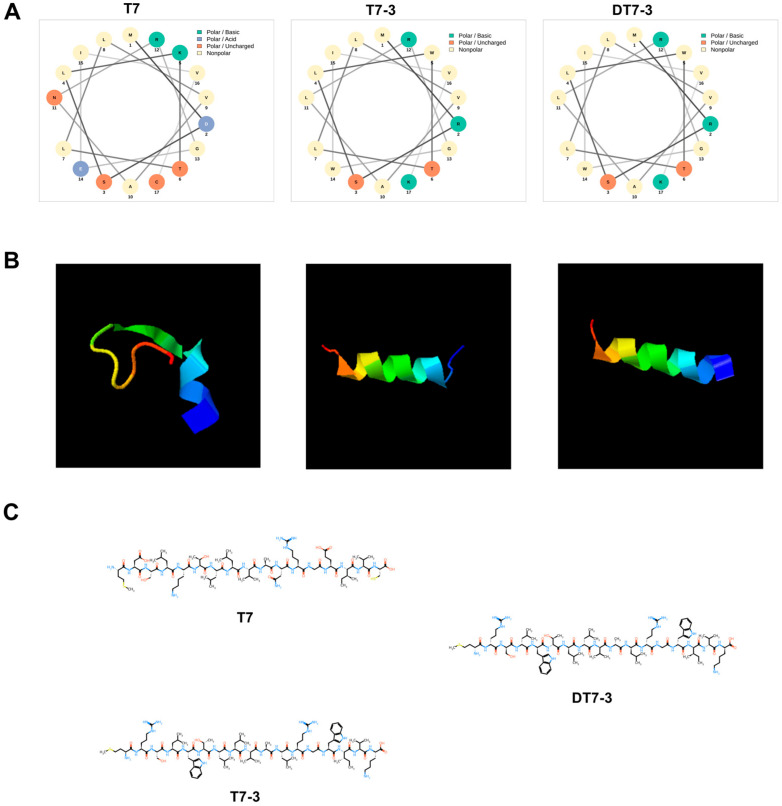
Characteristics of T7 derived peptides. (**A**) Helical diagram. (**B**) The predicted 3D model. (**C**) Planar structural formula. The primary sequences are as follows: T7: MDSLKTLLVANRGEIVC; T7-3: MRSLWTLLVALRGWIVK; DT7-3: mrslwtllvalrgwivk (lowercase denotes D-enantiomers).

The characterization of peptides provided crucial insights into this rational design. As predicted by I-TASSER (Figure 2B), the parental T7 peptide was dominated by an unstructured, random coil. In stark contrast, the engineered sequences of T7-3 and DT7-3 were predicted to fold into a stable α-helical conformation (Figure S2). This structural transformation is visually explained by the helical wheel projections (Figure 2A), which show that T7-3 possesses a near-perfect amphipathic structure, segregating its potent hydrophobic residues (F, W, L, V, I) to one face and its cationic residues (R, K) to the other—a feature absent in the disorganized T7. Furthermore, the planar structural formula (Figure 2C) visually confirms the successful incorporation of the two bulky indole side-chains from the Tryptophan (W) substitutions.

Combined with the physicochemical properties in Figure 1, these analyses strongly suggest that our rational design strategy successfully transformed a low-activity, unstructured peptide (T7) into a highly structured, amphipathic, and potently armed α-helical peptide (T7-3, DT7-3). This indicates the design holds significant potential for the enhanced biological activity subsequently observed.

### 3.2. High Prevalence of MDR in Clinical H. pylori Isolates

**Figure 3:**
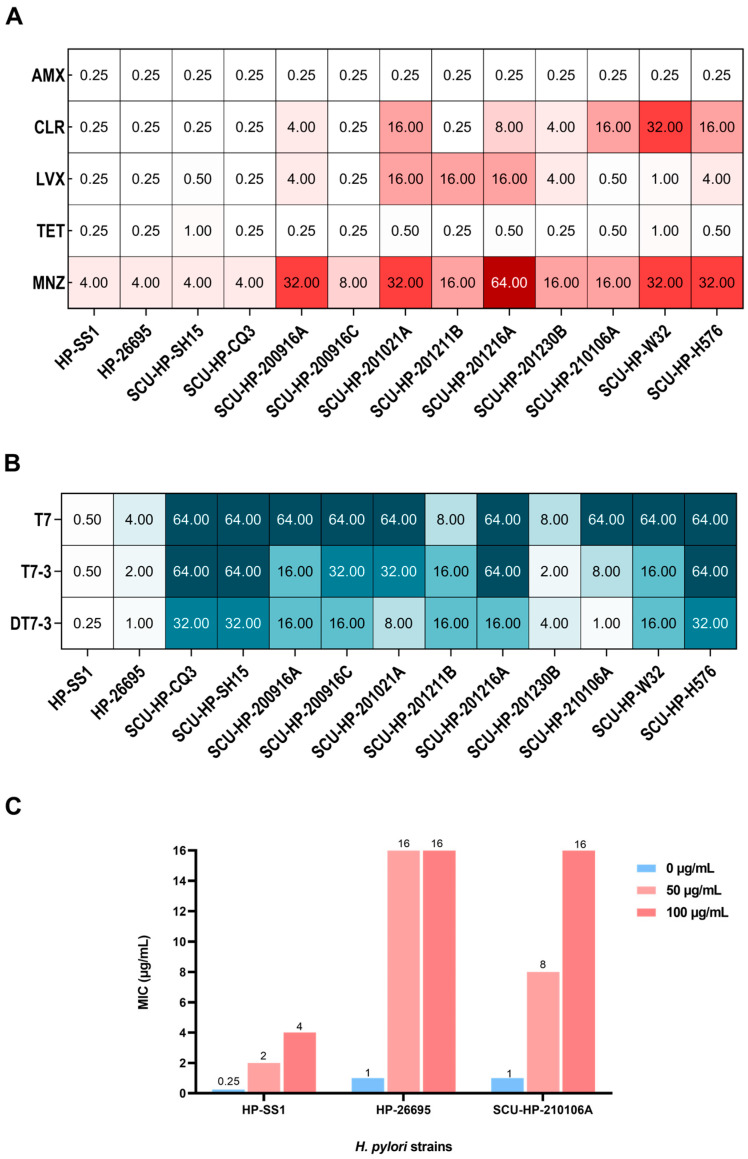
Susceptibility profiles and cholesterol resilience of *H. pylori* strains. (**A**) Antibiotic resistance heatmap of clinical (n = 11) and standard (n = 2) strains. Red intensity represents MIC values (μg/mL) within cells; darker red indicates higher resistance based on CLSI breakpoints. (**B**) Antimicrobial activity heatmap of peptides T7, T7-3, and DT7-3 against clinical isolates. Blue intensity reflects MIC values (μg/mL); darker blue signifies higher MICs (lower potency). The predominantly lighter profile of DT7-3 demonstrates its superior potency against diverse MDR phenotypes. (**C**) Impact of cholesterol on the antimicrobial activity of the lead peptide DT7-3. The MIC values against standard (*H. pylori* SS1 and 26695) and clinical MDR (*H. pylori* SCU-HP-210106A) strains were determined after a 12 h pre-incubation in media supplemented with 0, 50, and 100 µg/mL water-soluble cholesterol.

Clinical isolates were obtained to reflect the current resistance landscape (Figure S1). The antibiotic susceptibility profiles of the 11 clinical isolates and 2 standard strains were determined. As shown in Figure 3, the two standard reference strains (*H. pylori* 26695 and SS1) were highly susceptible to all five antibiotics tested, with MIC values consistently low (≤0.25 µg/mL for most, 4 µg/mL for MNZ). In sharp contrast, the clinical isolates exhibited extremely high levels of resistance. MIC values for clarithromycin (CLR) and metronidazole (MNZ) frequently reached 16~64 µg/mL, far exceeding the resistance breakpoints (0.5 µg/mL and 8 µg/mL, respectively).

The antibiotic susceptibility landscape depicted in Figure 3A illustrates a pronounced disparity between the pan-susceptibility of the standard reference strains and the pervasive resistance observed among the clinical isolates. Phenotypic analysis revealed that 90.9% (10/11) of the clinical cohort exhibited resistance to at least one antimicrobial agent. While single-drug resistance (SDR) and double-drug resistance (DDR) were each observed in 18.2% (2/11) of the isolates, a substantial majority (54.5%, 6/11) were classified as multidrug-resistant (MDR). Notably, this MDR group included the highly recalcitrant strain SCU-HP-W32, which demonstrated a quadruple-resistance profile (CLR-R, LVX-R, TET-R, and MNZ-R). These findings underscore the severe burden of multidrug resistance within our clinical isolates, validating their suitability for evaluating novel therapeutic interventions.

### 3.3. DT7-3 Exhibits Potent Activity Against MDR Strain

To evaluate the translational potential of the engineered peptides in the context of escalating antibiotic resistance, the antimicrobial susceptibility of 11 clinical *H. pylori* isolates was systematically profiled. As visualized in Figure 3B, the antimicrobial landscapes of the three peptides exhibited distinct stratification. The parental peptide T7 displayed negligible inhibitory activity across the entire panel, with MIC values consistently exceeding 64 µg/mL. While the modified L-enantiomer T7-3 showed a marginal increase in potency, its performance was highly heterogeneous and largely insufficient against strains harboring high-level resistance to conventional therapeutic agents. We further examined whether the incorporation of host-derived cholesterol compromises the efficacy of DT7-3. As visualized in Figure 3C, the addition of exogenous cholesterol led to a dose-dependent increase in MIC values across all tested strains. For instance, the MIC for the triple-resistant isolate SCU-HP-210106A shifted from 1 µg/mL to 16 µg/mL at the highest cholesterol concentration (100 µg/mL). Despite this 16-fold shift—which aligns with characteristic cholesterol-induced resistance patterns—DT7-3 remained highly effective under maximum cholesterol pressure (MIC ≤ 16 µg/mL). This indicates that the peptide’s bactericidal potency is resilient to the biophysical barrier established by a rigidified, cholesterol-rich membrane.

**Figure 4:**
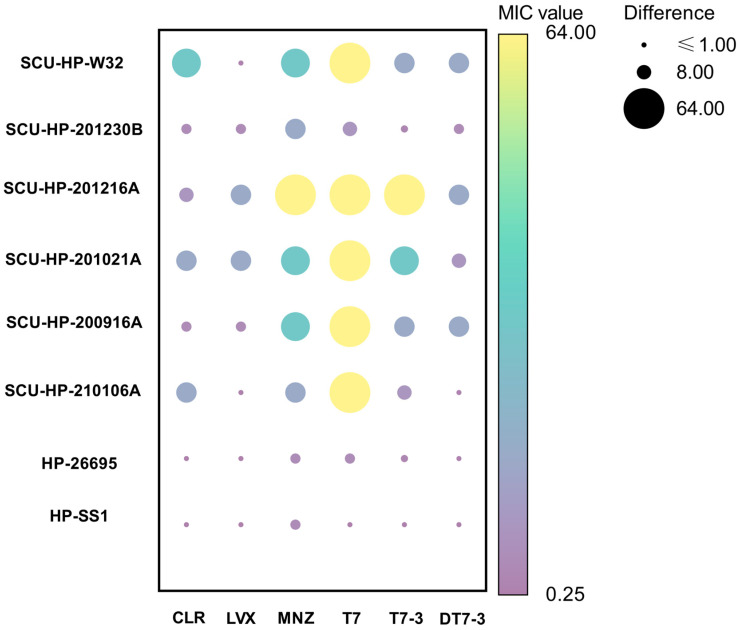
Comparative analysis of peptide activity against representative MDR *H. pylori* strains.

In contrast, the D-enantiomer DT7-3 demonstrated robust and uniform bactericidal efficacy across the clinical isolate panel. While the MIC values of DT7-3 (1–32 µg/mL) are numerically higher than those typically observed for amoxicillin in sensitive strains, they represent a clinically significant potency in the context of multidrug resistance. Crucially, the activity of DT7-3 remained consistent regardless of the underlying resistance genotypes. Isolates clinically resistant to Clarithromycin (CLR), Levofloxacin (LEV), and Metronidazole (MNZ), remained highly susceptible to DT7-3 (MICs of 1–8 µg/mL). A comparative analysis of representative multi-drug resistant (MDR) strains further highlighted this potency (Figure 4). Notably, against the triple-resistant isolate SCU-HP-210106A (CLR-R, LEV-R, MNZ-R), DT7-3 achieved a potent MIC of 1 µg/mL, representing a 64-fold enhancement in activity relative to T7. This superior performance suggests that while traditional small-molecule antibiotics are compromised by specific mutational adaptation, the D-enantiomer maintains its efficacy through direct, non-specific interaction with the bacterial lipid bilayer, supplemented by its enhanced proteolytic stability.

The generalized antimicrobial utility of DT7-3 was further corroborated by its efficacy against the Gram-positive resistant pathogen MRSA SCQL1 (Table S2). In contrast to the relative inactivity of T7, T7-3, and several first-line antibiotics (e.g., Erythromycin and Tetracycline), DT7-3 maintained a significant inhibitory effect with an MIC of 8 µg/mL. The ability of DT7-3 to maintain high potency across both Gram-negative and Gram-positive resistant models reinforces the hypothesis that it targets fundamental bacterial structures less prone to rapid mutational adaptation. Collectively, these data identify DT7-3 as a promising candidate for the eradication of highly recalcitrant *H. pylori* infections and potentially other MDR bacterial pathogens.

### 3.4. DT7-3 Possesses a Multifaceted Mechanism of Action

The clinical MDR isolate SCU-HP-210106A was selected as the model strain for all subsequent mechanistic evaluations, as it represents a challenging “triple-resistant” phenotype (CLR-R, LEV-R, and MNZ-R) commonly encountered in clinical eradication failures.

#### 3.4.1. Direct Membrane Disruption

**Figure 5:**
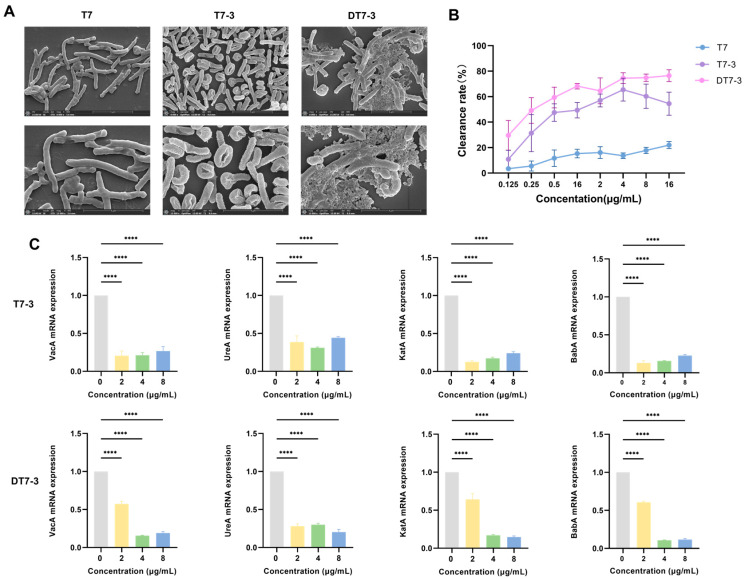
DT7-3 Possesses a Multifaceted Mechanism of Action. (**A**) SEM images of *H. pylori* treated with peptides. (**B**) Eradication of adherent *H. pylori* from GES-1 cells. (**C**) Relative mRNA expression of *H. pylori* virulence genes. **** *p* < 0.0001.

To visualize the morphological impact and correlate structural modifications with membrane-damaging activity, we treated *H. pylori* (SCU-HP-210106A) with all three peptides (Figure 5A). Bacteria treated with the parental peptide T7 were morphologically indistinguishable from the control, retaining their smooth surface, which is consistent with its lack of lytic activity. In contrast, treatment with T7-3 induced moderate morphological alterations, including surface roughening and wrinkling. The most severe damage was observed in the DT7-3 treatment group, where cells exhibited a complete loss of native morphology, severe shrinkage, collapse, and deep surface pitting. These observations confirm that bactericidal activity correlates directly with the ability to disrupt the *H. pylori* cell membrane (DT7-3 > T7-3 > T7).

#### 3.4.2. Eradication of Adherent *H. pylori*

We investigated the peptides’ ability to eradicate already adherent bacteria from GES-1 cell monolayers using the clinical MDR strain *H. pylori* SCU-HP-210106A (Figure 5B). The parental peptide T7 was ineffective (<20% clearance). In contrast, the modified peptides demonstrated significant clearance activity. T7-3 achieved ~65% clearance at 4 µg/mL. DT7-3 displayed the most potent eradication activity, achieving ~50% clearance at a low concentration of 0.25 µg/mL and reaching a plateau of ~75% clearance at 4 µg/mL. This demonstrates that DT7-3 can effectively disrupt and clear established *H. pylori* colonization.

#### 3.4.3. Downregulation of Key Virulence Genes

To elucidate the molecular basis for the anti-adhesion activity, we quantified the expression of key virulence genes in *H. pylori* SCU-HP-210106A after treatment with sub-inhibitory concentrations (Sub-MICs) of T7-3 and DT7-3 (Figure 5C). At these sub-lethal doses (2–8 µg/mL), where total membrane lysis was not yet predominant, both peptides exhibited significant suppression of core virulence factors. The L-peptide T7-3 exhibited potent suppression of all four virulence genes even at low concentrations (2 µg/mL). DT7-3 displayed a dose-dependent inhibitory profile. While showing moderate activity at 2 µg/mL, its potency increased sharply at 4 µg/mL and 8 µg/mL, strongly suppressing *babA* (adhesin), *katA*, and *vacA* (toxin) to levels comparable to or lower than T7-3. This indicates a “disarming” effect, where the peptide attenuates bacterial pathogenicity even at sub-lethal doses.

In summary, these results collectively demonstrate that DT7-3 targets *H. pylori* through a coordinated ‘triple-hit’ approach, sequentially impacting structural integrity, host–cell interaction, and virulence expression.

### 3.5. Evaluation of Biocompatibility and Selectivity

**Figure 6:**
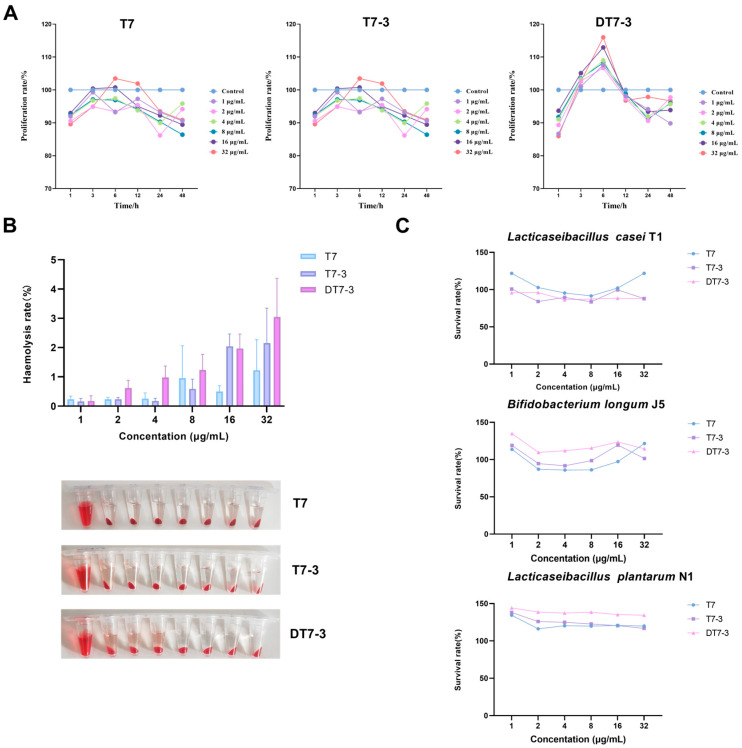
Biocompatibility and Selectivity of Peptides. (**A**) Cytotoxicity of peptides against GES-1 cells after 24 h incubation, assessed by CCK-8 assay. (**B**) Hemolytic activity of peptides against sheep erythrocytes. Triton X-100 (1%) and PBS served as the positive (100% lysis) and negative (0% lysis) controls, respectively. (**C**) Susceptibility of representative beneficial gut microbiota strains (*Lacticaseibacillus casei* T1, *Bifidobacterium longum* J5, and *Lacticaseibacillus plantarum* N1) to peptides.

To evaluate the clinical potential and therapeutic window of the peptides, the in vitro safety and selectivity were assessed against GES-1 and RBCs, as well as against representative beneficial gut microbiota strains (Figure 6).

In CCK-8 assays (Figure 6A), DT7-3 showed no significant toxicity toward human gastric epithelial cells (GES-1) at concentrations up to 8 µg/mL (>85% viability). DT7-3 exhibited negligible hemolytic activity against RBCs (<5% lysis) even at 32 µg/mL (Figure 6B). DT7-3 achieved an SI > 32 relative to the most sensitive MDR strain (MIC = 1 µg/mL), indicating excellent membrane selectivity. Crucially, DT7-3 showed no antimicrobial activity against beneficial gut microbiota. Even at the highest concentration tested (128 µg/mL), the growth of *Lacticaseibacillus casei* T1, *Bifidobacterium longum* J5, and *Lacticaseibacillus plantarum* N1was not inhibited (MIC > 128 µg/mL) (Figure 6C), yielding a gut microbiota-related SI > 16 (based on a median MIC of 8 µg/mL for *H. pylori*). Collectively, these quantitative indices define a wide therapeutic window for DT7-3, specifically targeting *H. pylori* while preserving host cells and commensal microbiota.

Collectively, these data define a wide therapeutic window for DT7-3, selectively targeting *H. pylori* while sparing host cells and the commensal microbiota.

## 4. Discussion

The rapid dissemination of multi-drug resistant (MDR) *H. pylori* has severely compromised the efficacy of standard eradication therapies, creating a global health imperative for novel therapeutic agents with distinct mechanisms of action [34]. In this study, we demonstrated that the rationally designed D-enantiomer peptide, DT7-3, possesses potent antimicrobial activity against a broad spectrum of clinical MDR *H. pylori* isolates, offering a promising alternative to conventional antibiotics [35]. The antimicrobial efficacy of DT7-3 is characterized by a synergistic ‘triple-hit’ mechanism. Firstly, it exerts rapid bactericidal action through direct membrane disruption. Secondly, at sub-lethal concentrations, the peptide significantly impairs the ability of *H. pylori* to adhere to gastric epithelial cells. Finally, it ‘disarms’ the pathogen by downregulating the expression of critical virulence genes, including *babA*, *ureA*, and *vacA*, thereby potentially reducing the risk of persistent infection and gastric damage.

The primary mechanism of action for most cationic antimicrobial peptides (AMPs) involves the initial electrostatic recruitment to the bacterial surface, followed by the irreversible disruption of the lipid bilayer [36]. Our SEM analysis provided direct morphological evidence for this process, revealing a clear structure–activity correlation. While the parental peptide T7, characterized by high proteolytic susceptibility and suboptimal amphipathicity, induced no discernible damage, DT7-3 triggered catastrophic membrane lysis and cellular collapse. This suggests that the D-amino acid substitution not only confers resistance to bacterial proteases but also optimizes the biophysical interactions required to perturb the bacterial envelope [10]. Unlike traditional antibiotics that target specific metabolic pathways, which are easily bypassed by single-point mutations, the physical membrane-lytic mechanism of DT7-3 presents a formidable barrier to the development of resistance [37]. Altering the fundamental composition and charge density of the entire bacterial membrane would impose a significant metabolic and energetic burden on the pathogen [38].

Beyond its efficacy against Gram-negative *H. pylori*, the remarkable inhibitory activity of DT7-3 against the Gram-positive resistant pathogen MRSA SCQL1 (MIC = 8 µg/mL) is a significant finding. However, the observation that DT7-3 spares beneficial Gram-positive beneficial gut microbiota like *Lacticaseibacillus* (MIC > 128 µg/mL) despite their shared Gram-staining characteristics warrants closer biophysical inspection. This selectivity likely stems from the fundamental differences in membrane phospholipid composition and cell wall architecture. Gram-negative *H. pylori* and pathogenic *S. aureus* membranes are typically rich in phosphatidylglycerol (PG) and phosphatidylethanolamine (PE), which provide high negative charge density and fluidity conducive to cationic peptide insertion [39]. In contrast, the membranes of certain beneficial *Lacticaseibacillus* strains often possess higher cardiolipin content and a thicker, more highly cross-linked peptidoglycan layer that acts as a physical and electrostatic sieve, potentially sequestering the peptides before they reach the lipid bilayer [40]. These results suggest that DT7-3 could serve as a versatile lead compound for developing next-generation broad-spectrum antimicrobials with refined species-selectivity [41].

Furthermore, our findings reveal a multifaceted anti-pathogenic profile of DT7-3 against *H. pylori*. The peptide demonstrated a significant capacity to eradicate adhered bacteria and, more importantly, to suppress the transcription of key virulence factors, including *babA*, *ureA*, and *vacA* [42]. A critical mechanistic question is whether these functional impairments are merely secondary consequences of membrane lysis. Notably, our results demonstrated significant anti-pathogenic effects at sub-MIC levels (0.5× and 0.25× MIC), where SEM observations confirmed that the bacterial cell wall remains largely intact despite initial surface perturbation [43]. This suggests a hierarchical mechanism of action: at sub-lethal doses, the primary “hit” involves physical interference, likely through steric hindrance or the displacement of surface adhesins, while the suppression of genes like *babA*, *ureA*, and *vacA* constitutes a secondary, stress-induced metabolic response [44,45]. The downregulation of *babA* (adhesin) and *ureA* (urease) suggests a potential to impair the initial colonization and acid-neutralizing capabilities of *H. pylori* in the hostile gastric environment [46]. Furthermore, the suppression of *vacA* indicates that DT7-3 might attenuate the induction of gastric epithelial vacuolation and subsequent tissue damage. This “virulence disarming” effect underscores the therapeutic potential of DT7-3 in mitigating pathogenicity even at concentrations that do not cause immediate cell death [9].

A critical challenge in the clinical translation of AMPs is their potential for non-specific systemic toxicity. DT7-3, however, exhibited an excellent safety profile with a high Selectivity Index (SI > 32 for RBCs) [25]. This high selectivity is likely governed by the fundamental differences in membrane composition; the peptide preferentially targets the anionic lipid-rich bacterial membranes (e.g., phosphatidylglycerol) over the zwitterionic and cholesterol-rich mammalian membranes [47]. Notably, a critical physiological factor influencing *H. pylori* pathogenesis is the availability of host-derived cholesterol. Unlike most bacteria, *H. pylori* possesses the unique capability to sequester cholesterol from the gastric mucosa and incorporate it into its plasma membrane via α-glucosylation. This biochemical modification significantly modulates membrane fluidity and establishes a biophysical barrier against traditional membrane-active AMPs [48,49]. Our targeted experimental verification confirmed this dependency, exhibiting an 8-to-16-fold shift in MIC values at 100 µg/mL cholesterol, which aligns perfectly with established models of cholesterol-induced resistance to endogenous AMP such as LL-37 [20]. Crucially, even with a modified, more rigid membrane, DT7-3 remains potent (maximum MIC = 16 µg/mL) against the MDR isolate. This resilience is indirectly substantiated by our biocompatibility profiles: DT7-3 maintains an exceptional Selectivity Index (SI > 32) against human erythrocytes—cells characterized by exceptionally high cholesterol-to-phospholipid ratios. The strong electrostatic attraction between the cationic peptide and the negatively charged components of the *H. pylori* surface appears sufficient to override the steric hindrance and increased packing density caused by cholesteryl glucosides. This empirical evidence confirms that DT7-3 maintains significant therapeutic potential in the cholesterol-rich environment of the human stomach. Interestingly, in cell proliferation assays we observed a transient increase in GES-1 metabolic activity at the highest concentration (128 µg/mL) after 6–12 h of incubation. This phenomenon is characteristic of a “hormetic response,” wherein cells undergo a compensatory metabolic burst and mitochondrial overactivity to counteract initial, low-level membrane-associated stress [50]. Such a response is typically a temporary defense mechanism that is eventually overwhelmed by 24 h, leading to the stabilized viability profiles observed later. Furthermore, the quantitative gap between the MICs for *H. pylori* and beneficial gut microbiota (SI > 16) represents a major advantage over conventional broad-spectrum antibiotics, which frequently cause severe gut dysbiosis and associated gastrointestinal side effects. This profile suggests that DT7-3 could function as a “precision” antimicrobial, eradicating the specific pathogen while preserving the homeostatic balance of the gastric and intestinal microbiota [51].

A notable limitation of the current study is the absence of direct proteolytic stability assays using purified host enzymes. The susceptibility of peptides to proteases, such as pepsin and trypsin in the gastric environment, remains a significant hurdle for their clinical application [52]. However, DT7-3 was specifically designed as an all-D-amino acid enantiomer to circumvent this challenge. Chiral inversion is a well-established strategy to render peptides resistant to enzymatic degradation, as natural proteases are stereospecific and generally fail to recognize or cleave D-peptide bonds [53]. While we did not perform kinetic stability tests in this work, the robust antimicrobial activity of DT7-3 in the presence of complex media indirectly supports its enhanced stability. Future investigations using simulated gastric and intestinal fluids will be essential to fully characterize its pharmacokinetic profile [12].

The antimicrobial landscape of *H. pylori* research includes several well-characterized AMPs, most notably the human cathelicidin LL-37 and amphibian-derived Magainin 2. While LL-37 has demonstrated potent bactericidal effects against *H. pylori* by targeting its membrane [54], its clinical application is fundamentally limited by its L-amino acid composition, which renders it highly susceptible to rapid degradation by gastric proteases such as pepsin [52]. In contrast, DT7-3, through its all-D-enantiomer configuration, circumvents this metabolic hurdle while maintaining equivalent or superior MIC levels (1–32 µg/mL) against a broad panel of multidrug-resistant clinical isolates. Furthermore, a comparison of safety profiles reveals that DT7-3 possesses a more favorable therapeutic window; its high selectivity (SI > 32) significantly exceeds that of LL-37, which is known to trigger pro-inflammatory responses and exhibit cytotoxicity toward certain host cells at bactericidal concentrations [55]. By combining the ‘triple-hit’ mechanism with exceptional proteolytic stability and probiotic-sparing selectivity, DT7-3 represents a more robust and clinically viable template for next-generation *H. pylori* eradication than traditional endogenous AMPs [56].

Despite these promising results, several limitations warrant further investigation. While the D-amino acid configuration is theoretically resistant to enzymatic degradation, its long-term stability in the presence of high-concentration pepsin and gastric acid remains to be experimentally quantified [57]. Secondly, although our in vitro data are robust, the complex physiological environment of the stomach, including the protective mucus layer and varying pH gradients, may influence the peptide’s bioavailability and activity. Future studies using in vivo infection models (e.g., C57BL/6 mouse models) are essential to validate the therapeutic efficacy, pharmacokinetics, and long-term safety of DT7-3 in a living system [58].

## 5. Conclusions

In conclusion, the D-amino acid peptide DT7-3 demonstrates potent, selective, and rapid bactericidal activity against clinical MDR-*H. pylori* strains. It operates via a novel multifaceted mechanism that combines rapid membrane lysis, eradication of adherent bacteria, and suppression of key virulence factors. The structural modifications in DT7-3 successfully achieved a high selectivity for pathogens over host cells or beneficial gut microbiota. Furthermore, DT7-3 maintains effective bactericidal activity, even in cholesterol-enriched environments. This favorable profile addresses a major bottleneck in AMP development and establishes DT7-3 as a robust candidate for further development as a novel therapeutic strategy to overcome *H. pylori* adhesion and drug resistance.

## Data Availability

The original contributions presented in the study are included in the article/Supplementary Material, further inquiries can be directed to the corresponding authors.

## Supplementary Materials

The following supporting information can be downloaded at: https://www.mdpi.com/article/10.3390/microorganisms14040744/s1, Figure S1: The mass spectrum of peptides; Figure S2: The predicted secondary structure of peptides; Figure S3: Isolation and identification of clinical *H. pylori* strains; Table S1: *H. pylori* primers sequences; Table S2: Peptide MICs against MRSA SCQL1.
