# Supplementary material for: Rationally Engineered D-Amino Acid Peptide DT7-3 Combats Multidrug-Resistant Helicobacter pylori via a Novel “Triple-Hit” Mechanism
Source: Microorganisms. 2026 Mar 26;14(4):744. doi: 10.3390/microorganisms14040744 (PMC13118709; doi:10.3390/microorganisms14040744)
Supplement: Supplementary file 1 [file microorganisms-14-00744-s001.zip › microorganisms-4148178-supplementary.pdf]

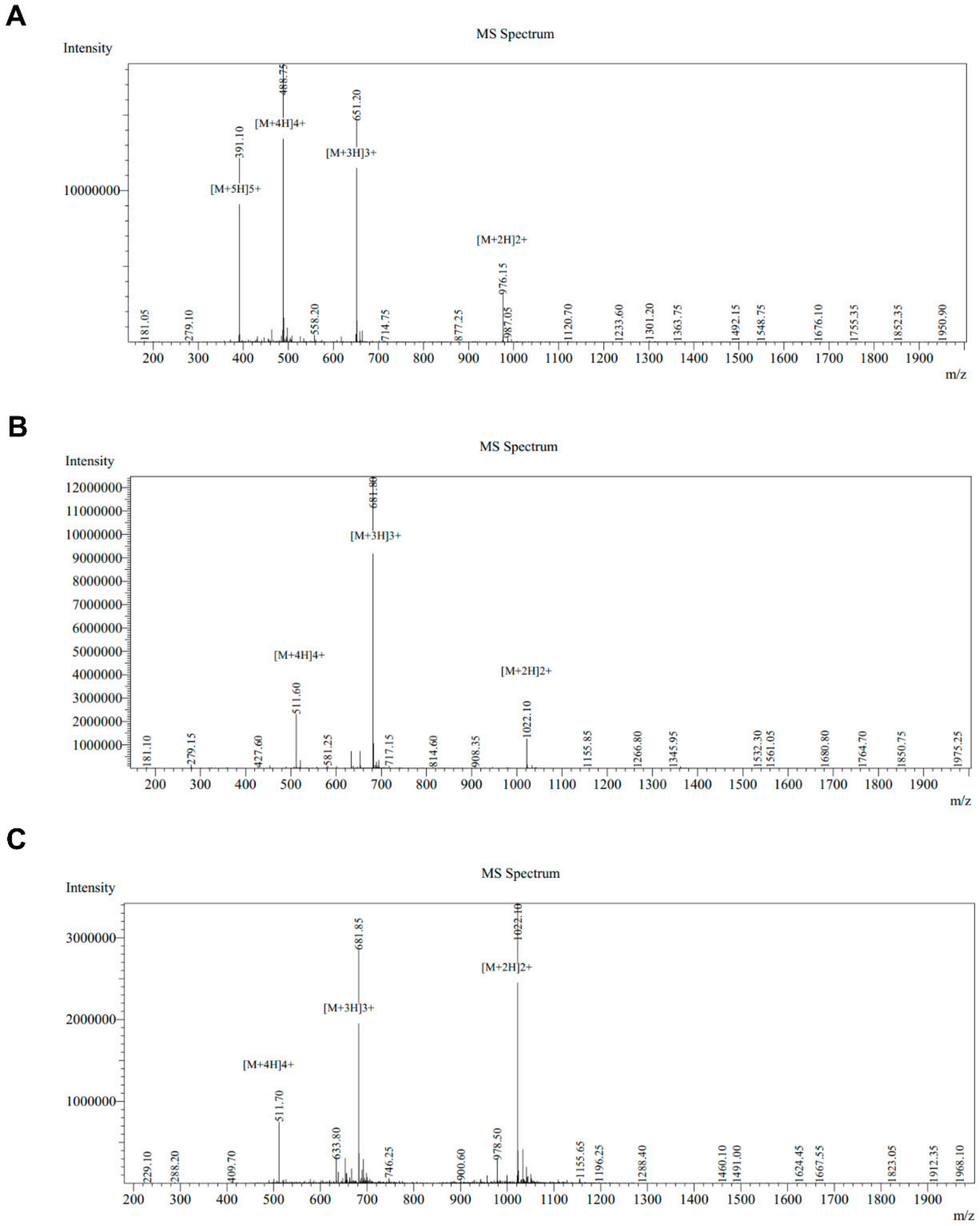

Figure S1 Mass spectrum of peptides. (A) T7. (B) T7-3. (C) DT7-3.

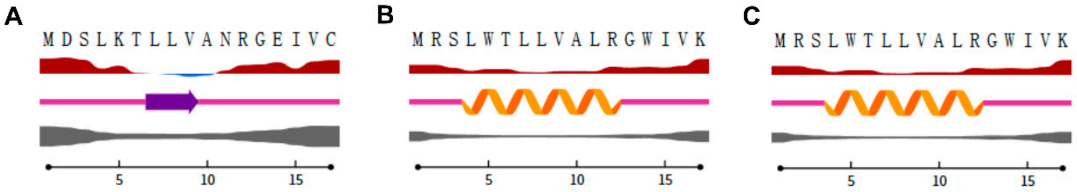

Figure S2 The predicted secondary structure of peptides.

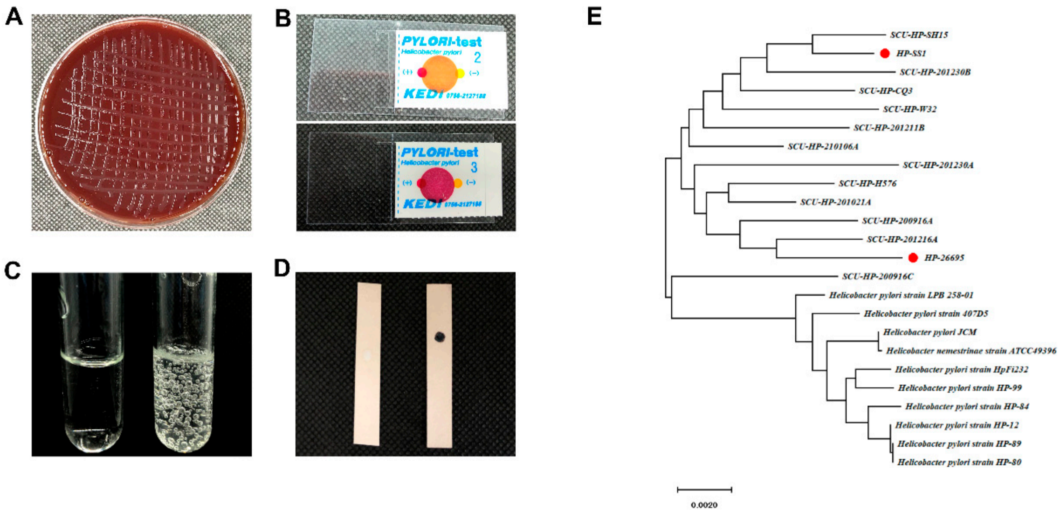

Figure S3 Isolation and identification of clinical *H. pylori* strains. (A) Typical colony morphology of *H. pylori* cultured for 3 days. (B) Rapid urease test. (C) Catalase activity test. (D) Oxidase activity test. (E) Phylogenetic tree constructed based on 16S rRNA gene sequences.

Table S1 *H. pylori* primers sequences.

Gene Name	Sequence (5' - 3')
16S rRNA-F	TAAGGAGGAGGAAGGTGGGG
16S rRNA -R	GCGATTCCAGCTTCATGCAG
VacA-F	GCTGGATTGGTGGTTTATGG
VacA-R	TGGTGGTTTCTTTGGTGGTT
UreA-F	TTGTCTGCTTGCCTATCAACCAAC
UreA-R	GAGCGGGACAGCGGTAAGG
KatA-F	TTCAACTCCACAATGCCCACTTC
KatA-R	AGTGATGCCAGAAGAAGATGCTAAG
BabA-F	AAGCCTATCAAATCCTCCAAACG
BabA-R	TGGCGAGCAGTTATTATTCCCT

Table S2 Peptide MICs against MRSA SCQL1.

Peptides/Antibiotics	MIC (μg/mL)
T7	256
T7-3	128
DT7-3	8
Erythromycin	256
Chloramphenicol	64
Tetracycline	128
Gentamicin	32
